# Rare Intradural Cervical Nerve Root Metastasis of Follicular Thyroid Carcinoma

**DOI:** 10.7759/cureus.898

**Published:** 2016-11-24

**Authors:** Joseph Keen, Elena Milosavljevic, George Hanna, Vadim Gospodarev, Ravi Raghavan, Samer Ghostine

**Affiliations:** 1 Department of Neurosurgery, Loma Linda University Medical Center; 2 School of Medicine, Loma Linda University Medical Center; 3 Department of Neuropathology, Loma Linda University Medical Center; 4 Department of Neurosurgery, University of California Riverside

**Keywords:** thyroid follicular carcinoma, nerve root metastasis, cervical spine, spinal metastasis, intradural metastasis, intradural tumor, nerve root, spine surgery

## Abstract

Intradural extramedullary nerve root metastasis is extremely unusual with only a handful of cases reported, and it presents most commonly in the thoracic and lumbosacral regions. We report the first case of metastasis to a ventral cervical nerve root in a patient with low-grade follicular thyroid carcinoma thought to be in remission for several years. Histopathology demonstrated malignant transformation and invasion of the nerve root. This case underscores that any history of malignancy regardless of staging, grading, or remission status should raise the suspicion of metastasis as it can mimic other spine and nerve sheath tumors and represent malignant transformation. Gross total resection can be safely achieved with intraoperative neuromonitoring and result in improved function; however, treatment is likely palliative.

## Introduction

Intradural nerve root metastases are extremely rare with only a handful of cases ever reported. The lumbar [1-4] and thoracic regions [5-6] tend to be more common areas of presentation, whereas cervical nerve root metastasis has been reported once for a case of breast cancer that spread via drop metastasis [7]. We report the first case of metastasis to and invasion of a ventral cervical nerve root in a patient previously diagnosed with low-grade thyroid carcinoma, who presented with right upper extremity radiculopathy and weakness after being in remission for several years. Patient consent was not necessary for this study as it was a retrospective study.

## Case presentation

### Presentation

A 61-year-old female with a history of follicular thyroid carcinoma, who underwent a total thyroidectomy followed by radioactive iodine (I-131) therapy nine years prior, presented with severe right-sided neck and upper extremity radicular pain as well as deltoid weakness that had progressed over six weeks. The patient denied sensory disturbance, bowel or bladder dysfunction, but needed a front-wheel walker when ambulating.

**Figure 1:**
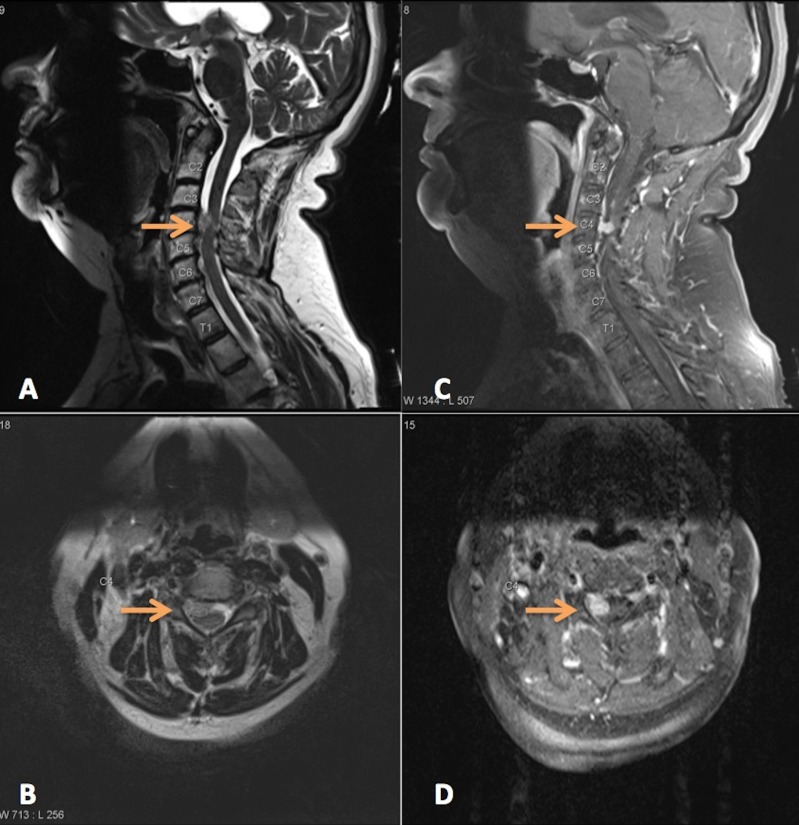
Preoperative MR Imaging A) Preoperative T2 sagittal MRI shows ventral C4-5 intradural mass w/ cord compression B) Preoperative T2 axial MRI shows right sided displacement of C5 nerve root C) Preoperative T1 sagittal MRI with contrast shows avid enhancement D) Preoperative T1 axial MRI with contrast shows avid enhancement

On examination, the only focal deficit was weakness in the right deltoid (2/5 strength). Magnetic resonance imaging (MRI) of the cervical spine revealed a ventral, intradural lesion at the C4-5 vertebrae that appeared to be compressing both the spinal cord and the nearby nerve roots (Figures 1A-1D). Initial impressions placed meningioma and schwannoma high on the differential, whereas metastatic thyroid cancer was considered less likely because of the uncharacteristic nature of papillary thyroid cancer to metastasize and the fact that the patient had been in remission for 10 years.

### Operative details

**Figure 2:**
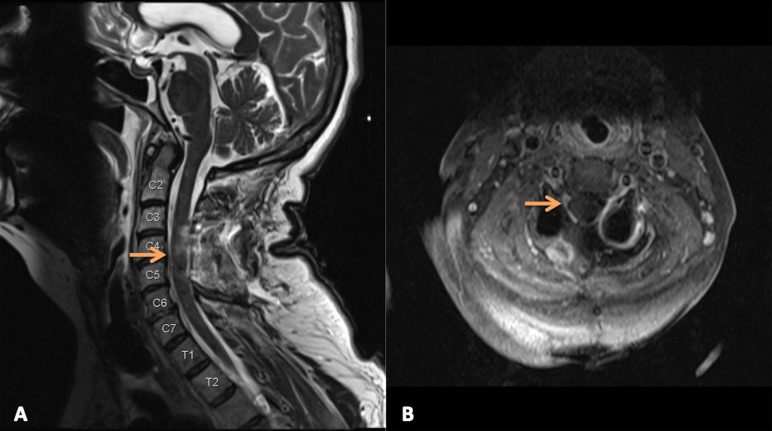
Postoperative MR Imaging A) Postoperative T2 sagittal axial MRI shows gross total resection and decompression of cord B) Postoperative T2 axial MRI shows gross total resection and decompression of cord

The patient underwent a posterolateral cervical approach for resection of the tumor, including C3-5 laminectomies followed by C3-C5 posterior instrumented fusion with lateral mass screws (left unilateral lateral mass screw at C4). Intraoperatively it was found that the tumor had clearly invaded the ventral C5 nerve root, but resection was possible without changes in neurophysiologic monitoring–somatosensory evoked potential (SSEP), motor evoked potential (MEP), electromyography (EMG). A postoperative MRI demonstrated gross total resection (Figures 2A-2B).

### Histopathology

**Figure 3:**
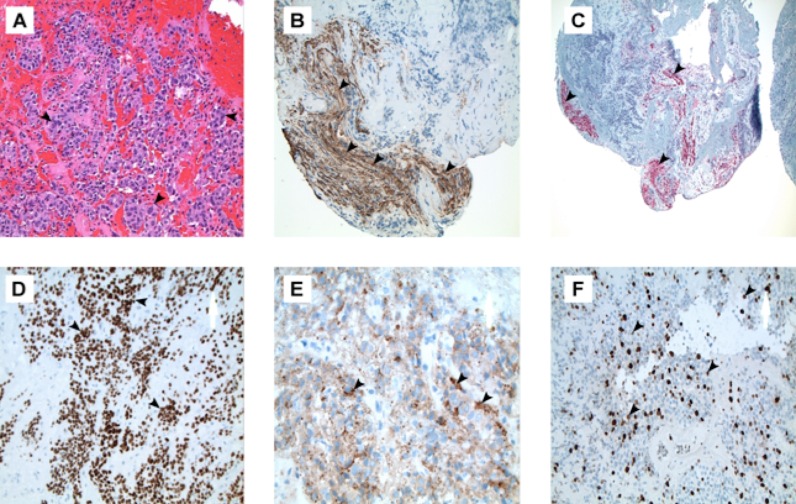
Histopathology A) H&E stain reveals poorly differentiated cells with mild nuclear pleomorphism (arrowheads) suggestive of carcinoma B) NFP (neurofilament protein) immunostaining (arrowheads) highlights invasion of nerve fibers C) S-100 immunostaining (arrowheads) confirms nerve infiltration D) TTF-1 (thyroid transcription factor) immunostaining (arrowheads) suggests thyroid origin E) Immunostaining for thyroglobulin (arrowheads) confirms metastasis of thyroid origin F) High Ki-67 labeling indices (arrowheads) support malignant transformation

Hematoxylin and eosin (H&E) staining revealed poorly differentiated cells with mild nuclear pleomorphism suggestive of metastatic carcinoma with both follicular and anaplastic features (Figure 3A), whereas positive staining with neurofilament protein (NFP) (Figure 3B), and S-100 (Figure 3C) confirmed invasion of nerve fibers. Staining with thyroid transcription factor 1 (TTF-1) (Figure 3D) and thyroglobulin (Figure 3E) confirmed thyroid origin. And, finally, high Ki-67 labeling indices suggested malignant transformation (Figure 3F).

### Postoperative course

Postoperatively, right deltoid strength improved from 2/5 to 3/5 strength with good radicular pain relief. Her thyroid-stimulating hormone (TSH) remained persistently elevated at 19.2 uIU/mL (normal 0.8-7.7 uIU/mL) and she was started on levothyroxine 75 mcg daily. Several weeks later, she was readmitted and treated with an oral dose of 218 millicuries radioactive iodine (I-131) and her levothyroxine was temporarily stopped. After the patient was determined to have safe body radiation levels of 5.53 mR/hr she was discharged home. A whole-body radioactive iodine (I-131) scintigraphy on post-therapy days one and 14 indicated multiple foci of increased I-131 uptake in the left submandibular gland, salivary glands, cranium, thorax, pelvis, liver and proximal thighs, without any uptake in the thyroid. The patient’s diagnosis was advanced to Stage 4 and, unfortunately, she died eight months later due to disease progression.

## Discussion

Intradural spinal metastasis with invasion of a nerve root is extremely unusual and has been rarely reported. It has been reported mainly in the thoracic [5-6] and lumbar [1-4] regions with one case of breast adenocarcinoma in the cervical spine [7]. Although there is a report of papillary thyroid carcinoma metastasizing to a thoracic nerve root in a patient with known cervical lymph node and sacroiliac joint spread several years following initial multi-modal therapies [6], our case is unique because it involves intradural metastasis to a ventral cervical nerve root of follicular thyroid carcinoma as a first sign of metastasis in a patient considered to be in remission for 10 years. To our knowledge, this would be the first reported case of follicular thyroid cancer with malignant transformation that metastasized to a ventral cervical nerve root.

Follicular thyroid carcinoma is considered low-grade with high survival and cure rates and a low propensity for metastasis [8]. When it does metastasize, it is typically found in the lungs and bone. Various routes of dissemination have been proposed, which include hematogenous, lymphatic, spinal fluid, direct invasion, and retrograde endoneurial spread, which has been described for head and neck cancers [5]. Our case was likely due to either hematogenous or retrograde endoneurial spread as opposed to local expansion from a vertebra or via spinal fluid pathways as the patient did not have any adjacent bone or brain involvement, respectively.

The first reported incidence of intradural, extramedullary nerve root metastasis was in 1977 from an autopsy series by Johnson, et al. [4], who identified two patients out of 500 with lumbar dorsal root ganglia metastases, who had prior histories of pulmonary oat-cell and renal cell cancer. Subsequently, a handful of nerve root metastases have been reported in the thoracic to sacral regions. Only one case of cervical nerve root metastasis has been reported for breast adenocarcinoma, which has a higher propensity for metastasis [7]. However, this most likely occurred via the spinal fluid pathway as the patient had intra-cerebral metastasis.

In general, nerve root and/or dorsal root ganglia metastases mimic radiculopathy and/or cauda equina syndrome, with the earliest symptoms being pain and weakness, followed by sensory loss, and bowl and bladder dysfunction [6]. According to Jung, et al., pain is the presenting symptom in 90–95% of patients [5], which was manifest in our patient, who first developed right-sided neck and radicular upper extremity pain followed by gradual progression of deltoid weakness.

Intradural metastases are indicative of advanced and extensive malignancy and therefore poor prognosis [8]. They are rarely curable because of the infiltrative and progressive nature of the primary tumor with an average life expectancy of three to four months after discovery of the intradural metastasis [9]. Most cases of intradural metastasis are treated with palliative radiation [8-9]; however, it has been suggested that surgical removal should be considered for patients with intramedullary spinal cord metastases, especially low-grade thyroid types, as median survival times have been doubled compared to conservative management [9]. A recent review of spinal metastasis in thyroid cancer suggests that the prognosis may be better when compared to other tumors that spread to the spine; and furthermore, patients were found to have improved survival if they were able to have complete metastasectomies versus palliative resection [8]. Jeon, et al. reported a case of intradural, intramedullary spinal cord metastasis from papillary thyroid carcinoma in a 44-year-old woman who presented with bilateral lower leg pain 12 years after being treated with total thyroidectomy [9]. The patient survived for two years after surgical resection of the tumor. Another case reported by Winkelman, et al. [10] describes a 45-year-old male who developed Brown-Sequard syndrome secondary to a solitary intradural, intramedullary spinal metastasis of mixed papillary and follicular thyroid cancer between the fifth and seventh cervical vertebrae. The tumor was resected and the neurological symptoms abated. The patient was subsequently treated with radioactive iodine and radiation to the whole spine without recurrence at three years.

Although our patient succumbed to her disease at eight months post-resection, her quality of life was improved as the surgery alleviated her radicular pain and slightly improved her dominant arm weakness. The decision for surgery and surgical approach should always be tailored to each patient’s individual oncological scenario, including pathology, location, staging, metastatic disease burden, and life expectancy, but early resection can help minimize neurological deficits and improve the quality of remaining life [8-10]. Cervical nerve root metastases may be more amenable to surgery as they tend to involve solitary nerve roots as opposed to the lumbar or cauda equina roots where metastases can clump together multiple roots. Patients may also achieve better symptom relief as cervical metastases will most likely compress both nerve root and the spinal cord. In the case of nerve root invasion, we demonstrate that gross total resection can be safely achieved by using microsurgical technique and neurophysiological monitoring. However, complete recovery of function may be limited due to chronic injury caused by invasion. A posterolateral cervical approach followed by instrumented fusion allowed adequate access to and safe resection of the ventral point of adherence to the nerve root. The approach should provide best access with least disruption to normal structures to minimize neurologic injury and spinal instability.

As with any metastasis, the primary neoplasm should be treated in accordance with standard oncological protocol. For follicular thyroid cancer, a total thyroidectomy is required followed by adjuvant radioactive iodine therapy (I-131) and long-term TSH suppression with levothyroxine. Follow-up radioactive iodine (I-131) scintigraphy is used to evaluate the distribution of and possible recurrence of metastasis.

## Conclusions

Intradural metastases are rare and cervical nerve root invasion even rarer, especially for low-grade follicular thyroid carcinoma in a patient considered in remission for several years. This is the first report of follicular thyroid carcinoma with malignant transformation that metastasized to and invaded a ventral cervical nerve root. Any history of thyroid carcinoma regardless of staging, grading, or remission status should raise the suspicion of metastasis as it can mimic other spine and nerve sheath tumors and represent malignant transformation. Gross total resection can be safely achieved with intraoperative neuromonitoring and result in improved function; however, treatment is likely only palliative.
